# Intervention of the occlusion relations in the appearance of the modifications in sagittal and transversal plan in the anomalies of the class Ⅲ angle

**Published:** 2010-08-25

**Authors:** A Temelcea, I Simion, R Stanciu, D Stanciu

**Affiliations:** * >Clinic of Orthodontics ODF, Faculty of Dental Medicine, UMF ‘Carol Davila’ BucharestRomania; ** >Dental office S.C. Leodent SRL, BucharestRomania

**Keywords:** occlusion relation, maxillary development, mandibular development

## Abstract

During the development of the neuronic and visceral cranium, an important number of factors, not only genetic, but also functional, led to the apparition of some different aspects of growing at the level of each section: the basis of the cranium, the basis of the maxillary, dental alveolus, dental arch.

All these ‘aggravating’ or ‘compensatory’ changes are added to the level of the occlusion plan, leading to the establishment of the occlusion relations in the three plans. From now on, the occlusion will directly influence the development at the level of the above-described sections.

The upper maxillary, attached to the plan of the cranium, depends on it not only in the sagittal plan, but also in the transversal plan, and, indirectly, in the vertical plan. The shortening of the cranium plan leads not only to the posterior positioning of the maxillary, but also to its posterior rotation and the installation of a deficiency of vertical front development. 

It is obvious that the anatomical conditions which determine the growing direction down and in front (with the help of the stitch), such as the fixation on the front section of the cranium basis (on the plan), on each side of the midline, through the exterior ends of the frontal eminences and the support on the lower and distal section on the pterygoid processes of the sphenoid bone, introduce a growing component with a remarkable functional influence. 

The mandibula, established on the basis of the cranium (on the clivus) with the help of the temporomandibular joint, depends more on the genetic factor of growing than on the functional one and the condylar growing represents a pattern of a very fast adaptation to the type of the facial rotation.  

In these conditions, the mandibular position in the interior of the maxillary arch will have as a consequence the radiar stimulation of the upper maxillary and the control of the development in radiar plan of the mandibula.

The reverse of the transmission with the help of the occlusion determinatives, of the vertical forces determined by the mobile musculation of the mandibula, will deprive the upper maxillary of the functional stimulus of development, and, the mandibula will not follow anything else but the genetic pattern of development.

The installation of the reversed inter–maxillary deviation from a cause or another generates a clinical image with particular aspects, which presents a common element, the reverse of transmission of occlusal forces.

In this context, the alveolar–dental processes, first submitted to the functional occlusion factor, develop two patterns of growing, one aggravating and the other one compensatory (Figure 1).

The aggravating pattern is characterized by the absence of development or the palatal declivity of the upper dental–alveolar process, with changes in the reversed way at the lower arch, a fact that determines an excess of growing in the lower part and a deficit of growing in the upper part. (Figure 1, left). 

**Figure 1:**
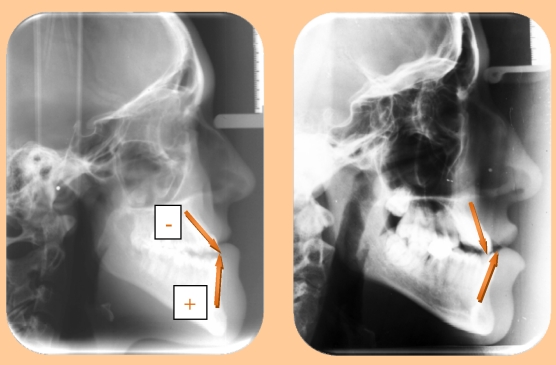
Profile teleradiography of a patient with malocclusion of the 3rd class and a pattern of compensatory growing (left) and the profile teleradiography of a patient with malocclusion of 3rd class and a pattern of aggravating growing (right);  the arrows show the way of development of the alveolar processes in the maxillary and mandibulary part

What  is characteristic, it is a reduced deviation in the sagittal plan between the two bases, and the functional factor plays an important role. 

The pattern of compensatory growing of the alveolar processes is determined by a vestibular development of the upper dental–alveolar process and a lingual development of the lower dental–alveolar process, the functional factor (lips and tongue) trying to compensate the large deviation between the bases of the maxillary. Moreover, the genetic factor plays an important role.

Certainly, between the two extremes, there is a large variety of clinical situations, which are also determined by the moment of the interception of the dental-maxillary anomaly. However, the occlusion factor plays an important role in the installation of the deviation between the bases, smaller at the compensatory pattern and determinative in the case of the aggravating pattern. (Figure 2).

**Figure 2:**
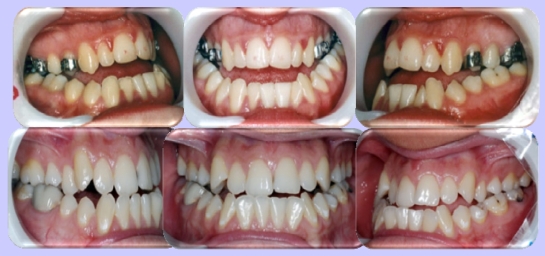
Occlusal aspect of a patient with compensatory pattern of growing (up) and aggravating (down)

Of course, the therapy of the anomaly is connected to the two patterns mentioned above. If in the case of the aggravating pattern the chances for the orthodontic energetic therapy (orthodontic urgency) are numerous, in the other form, the orthodontic–surgical therapy has an important role.  

In all forms of occlusion, the occlusion relations play an important role in the final aspect in malocclusion and more importantly in the installation of the troubles from the third class, with the reverse of the anterior guidance.  
